# 5G Indoor Positioning Error Correction Based on 5G-PECNN

**DOI:** 10.3390/s24061949

**Published:** 2024-03-19

**Authors:** Shan Yang, Qiyuan Zhang, Longxing Hu, Haina Ye, Xiaobo Wang, Ti Wang, Syuan Liu

**Affiliations:** 1 China Unicom Smart City Research Institute, Beijing 100102, China; yangs72@chinaunicom.cn (S.Y.); zhangqy383@chinaunicom.cn (Q.Z.); hulx31@chinaunicom.cn (L.H.); yehn3@chinaunicom.cn (H.Y.); wangxb161@chinaunicom.cn (X.W.); wangti2@chinaunicom.cn (T.W.); 2 Academy for Network and Communications of CETC, Shijiazhuang 050081, China

**Keywords:** 5G indoor positioning, indoor positioning error correction, neural network

## Abstract

With the development of the mobile network communication industry, 5G has been widely used in the consumer market, and the application of 5G technology for indoor positioning has emerged. Like most indoor positioning techniques, the propagation of 5G signals in indoor spaces is affected by noise, multipath propagation interference, installation errors, and other factors, leading to errors in 5G indoor positioning. This paper aims to address these issues by first constructing a 5G indoor positioning dataset and analyzing the characteristics of 5G positioning errors. Subsequently, we propose a 5G Positioning Error Correction Neural Network (5G-PECNN) based on neural networks. This network employs a multi-level fusion network structure designed to adapt to the error characteristics of 5G through adaptive gradient descent. Experimental validation demonstrates that the algorithm proposed in this paper achieves superior error correction within the error region, significantly outperforming traditional neural networks.

## 1. Introduction

In outdoor environments, GPSs (Global Positioning Systems) can provide high-precision positioning services [1]. However, within indoor environments, satellite positioning signals are often blocked, making it challenging to obtain positioning information through satellites. Consequently, indoor positioning technologies [2] such as Wi-Fi [3,4], Bluetooth [5], UWB [6], and 5G positioning [7] have emerged. Nevertheless, the field of indoor positioning still faces several unresolved issues. For instance, there exist numerous standards for indoor positioning technologies, resulting in a lack of a unified specification system. Moreover, the deployment costs of equipment are high, leading to increased operation and maintenance expenses. Utilizing 5G equipment, originally deployed for communication purposes, for positioning can potentially address these challenges effectively.

Amid the rapid development of the mobile network communication industry, the widespread adoption of 5G in the consumer market has notably addressed the indoor coverage issue by deploying cost-effective and adaptable 5G base stations [8]. Leveraging the advantages of the communication network, indoor positioning technology efficiently harnesses 5G signals to enable indoor positioning functionality, eliminating the need for redundant positioning network deployments. The amalgamation of 5G base stations with indoor positioning technology maximizes the utilization of the communication network [9,10,11]. Reference [12] utilized 5G signal millimeter-wave technology for precise positioning, leveraging Device-to-Device (D2D) communication technology to enhance collaborative positioning capabilities. It employed reinforcement learning methods to select the appropriate positioning approach for each signal, achieving centimeter-level localization accuracy. Reference [13] employed 5G positioning reference signals to estimate path delays and achieve receiver time synchronization, enabling precise localization through time-of-arrival (TOA) methods.

Although 5G technology has made significant advances in positioning compared to previous technologies, we still face the challenges of positioning errors in practical applications. These errors may stem from various complex factors, including but not limited to signal multipath effects, building obstructions, weather conditions, and more. These factors negatively impact the accuracy of 5G positioning, making it essential to delve into the mechanisms behind positioning errors and explore effective correction methods to enhance the reliability and precision of 5G positioning services.

In this article, a backpropagation neural network for 5G positioning error correction is proposed based on an adaptive global gradient descent algorithm. The method aims to correct errors generated in indoor environments during 5G positioning. This research primarily focuses on the following scenario: In indoor scenarios, corrections are applied to positioning coordinates with errors generated by existing 5G positioning methods, such as AOA [14] positioning, utilizing available information. This approach is taken when it is not possible to obtain measurement information, such as signal propagation time or angles, through closed-source devices, making it challenging to enhance the positioning accuracy further. The goal is to achieve rapid positioning and direct correction using existing methods and devices.

The main contributions made by the content studied in this paper are as follows:

(1) We analyzed a dataset constructed for indoor scenarios to identify the primary characteristics causing 5G positioning errors in indoor environments.
(2) Addressing non-linear features such as noise and multipath interference prevalent in indoor scenarios affecting 5G positioning errors, we introduced a neural network-based 5G positioning error correction network. The network effectively reduces the positioning error.
(3) In response to the causes of errors in indoor scenarios, we enhanced and optimized neural networks. We proposed a multi-level fusion network structure based on adaptive gradient descent. The network structure effectively prevents falling into local optima in this scenario.

The rest of the paper is organized as follows. Section 2 introduces the principles of 5G NR positioning, related research on wireless signal positioning error correction, and relevant methods for positioning error correction. Section 3, based on the AOA positioning principle, presents a dataset from real-world scenarios and analyzes the characteristics of 5G positioning errors in that environment. Section 4 describes the research on the 5G-PECNN algorithm, improvements to the designed model, and details of algorithm optimization methods. Section 5 demonstrates experimental testing of the designed 5G-PECNN algorithm using the dataset and analyzes and discusses the algorithm’s performance. Section 6 summarizes the article and discusses the advantages and limitations of this approach and future work.

## 2. Related Work

The objective of this section is to conduct an investigation into feasible correction methods for 5G positioning errors, aiming to obtain reasonable techniques for correcting 5G positioning errors. Currently, there is relatively limited direct research on 5G positioning error correction. We draw guidance from various related research areas, including the principles of 5G positioning, correction studies on similar wireless signal positioning errors, and correction methods, to form a framework for 5G positioning error correction.

### 2.1. Research on 5G NR Positioning Principles

5G NR (New Radio) positioning is a method in the new generation of mobile communication technologies, categorized into geometric and fingerprint positioning.

5G NR fingerprint positioning [15] is an indoor method leveraging signal characteristics for precise user equipment location determination. It relies on Received Signal Strength Indication (RSSI) [15] and Channel State Information (CSI) [16], with RSSI measuring signal power and CSI providing detailed channel information. Fingerprint positioning entails collecting and storing signal fingerprints from various locations to create a database. Real-time signal fingerprints are compared with the database to infer location, employing machine learning and deep learning techniques [17] to learn signal-location relationships.

Geometric positioning involves measuring distance or angle information between a target and multiple base stations, using methods like TOA, TDOA, and AOA.

TOA (time of arrival) [18] determines distance by measuring signal propagation time from transmitter to receiver, crucial for 5G NR distance measurement. RTT [19] measurements are common for practical scenarios.

TDOA (Time Difference of Arrival) [15] determines position by measuring time difference of signal arrival at different receivers, using hyperbolic curves to solve for the user equipment’s location. In 5G NR, it includes OTDOA (Observed Time Difference of Arrival) and UTDOA (Uplink Time Difference of Arrival) based on signal direction.

AOA (angle of arrival) [15] determines position by measuring the angle at which the signal arrives at the receiver, achieved with directional antennas or antenna arrays. In 5G NR, base stations with antenna arrays enable user equipment positioning by measuring the angle of signal arrival. The AOA-based method only requires accurate measurement of the incoming direction of the received signal, bypassing the need for precise time measurement in TOA/TDOA positioning methods. Millimeter-wave communication technology and massive MIMO have excellent directionality and higher resolution beams, resulting in higher accuracy in angle of arrival measurements. Methods for estimating arrival angles using antenna arrays mainly include MUSIC (Multiple Signal Classification) [20], beamforming [21], and the ESPRIT algorithm (Estimation of Signal Parameters via Rotational Invariance Techniques) [22], etc.

In the field of AOA positioning, reference [23] proposed an arrival direction estimation method based on the Kronecker subspace. This method employs a new formula using Khatri–Rao product array processing and can handle up to N − 1 coherent signals using an array with N sensors. The performance of this method was compared with traditional algorithms through computer simulation results. This method is named Kronecker–MUSIC. Reference [24] proposes a new framework integrating deep learning into massive MIMO systems for channel estimation and angle of arrival estimation. Deep neural networks (DNNs) are utilized for both offline and online learning, acquiring statistical information of wireless channels and spatial structures in the angle domain to enhance positioning accuracy. Reference [25] proposes a method for extracting arrival angles (AOA) and arrival times (TOA) from Channel State Information (CSI) using the MUSIC algorithm. It investigates the use of frequency hopping and smoothing techniques to enhance AOA and TOA estimation. The improved MUSIC method is compared with traditional MUSIC methods in AOA and TOA estimation applications, where CSI is collected in real-world environments and phase errors are mitigated through interpolation. Reference [26] proposes a single-base station positioning method based on 5G signals, which jointly estimates the arrival angle (AoA) and time of flight (ToF) for each path, achieving centimeter-level positioning accuracy. Reference [27] demonstrates the uniqueness of estimating AoA and AoD using random beamforming beam training. The impact of training length on the paired error probability of AoA and AoD is studied. The paper introduces an iterative positioning algorithm to estimate the position of mobile terminals, showing centimeter-level positioning accuracy. Furthermore, it proposes a method to enhance the estimation of path parameters (AoA and AoD) based on the geometric relationship of multiple terminal (MT) positions and suggests a blocking estimation method by comparing the maximum likelihood estimation of path loss and the path loss estimation based on MT positions.

### 2.2. Neural Network-Based Positioning Error Correction

Many different positioning error correction methods have been proposed in the literature.

First, methods for improving positioning accuracy by constructing transformation formulas through mathematical modeling have been proposed. Reference [28] introduces an innovative indoor positioning technology focused on improving node localization accuracy and stability indoors; it utilizes a novel RSSI distance prediction and correction model, incorporating correction factors and an enhanced centroid localization algorithm. By leveraging low-energy Bluetooth Low Energy (BLE) beacons, the technology notably reduces average positioning error and enhances stability, achieving significant accuracy improvements such as an 80.97% increase in office settings.

Second, there are methods for improving positioning accuracy through enhanced filtering techniques. Reference [29] proposed a particle filtering localization method utilizing LoRa RTT (Round-Trip Time) ranging to address challenges in Indoor Positioning Systems (IPSs). The method involves LoRa pseudorange fitting for calibration, aiming to enhance the accuracy of indoor positioning. Reference [30] presents a Downlink Angle of Departure (DL-AOD) localization algorithm, refining UE coordinate estimation through channel measurements. By optimizing DL-AOD solutions via spatio-temporal filter coefficient adjustments and introducing a reliability function to account for Non-Line-of-Sight (NLOS) propagation effects, the algorithm excels in sub-meter-level positioning accuracy, notably improving performance in NLOS environments.

Third, methods for improving positioning accuracy by integrating additional positioning signal information have been proposed. Reference [31] proposed a method to improve indoor positioning accuracy by combining inertial navigation with environmental adjustments. This involves digitizing spatial settings, estimating coordinates through inertial navigation, and iteratively refining positioning for increased precision. Reference [32] proposed a method to enhance positioning accuracy in non-line-of-sight (NLoS) scenarios using integrated Wi-Fi devices and ultra-wideband (UWB) ranging systems. It introduces inertial measurement units (IMUs) to calibrate UWB ranging, significantly reducing average ranging errors.

In addition to the methods mentioned above, there is also a neural network-based approach for correcting positioning errors. Positioning coordinate error correction can be viewed as an end-to-end multi-input multi-output regression task from coordinates with errors to corrected coordinates. Neural networks exhibit significant advantages in regression tasks [33]; their nonlinear modeling capability enables them to capture nonlinear relationships in complex data more accurately, while their flexible structure design allows adaptation to various types and scales of regression problems. Furthermore, neural networks can automatically learn feature representations from input data without relying on manually designed feature engineering, thereby improving model generalization and performance. Additionally, neural networks have the ability to handle large-scale data and support end-to-end learning, eliminating the need for manual intervention in the computation process from the raw data to the final output, thereby making model training more efficient. Neural networks are a powerful tool in regression tasks and have been widely applied with success in many fields [34,35,36]. Their advantages not only lie in efficient modeling and processing of data but also in their flexibility and ability to automatically learn features, providing robust support for regression analysis.

A neural network is a computational model inspired by the human brain’s neural system, aiming to simulate the information transmission and processing among neurons. It consists of neurons (or nodes) and the weights connecting these neurons, organized into hierarchical layers typically including input, hidden, and output layers.

Neurons are the basic units of neural networks, receiving inputs from other neurons and producing outputs. Each neuron has an activation function, which weights the input signals, sums them up, and generates an output. Common activation functions include Sigmoid, ReLU (Rectified Linear Unit), and Tanh. Connections between neurons are represented by weights, with each connection having a corresponding weight value to adjust the importance of input signals. Additionally, each neuron has a bias term to adjust the input range of the activation function. Weights and biases are parameters of the neural network that need to be learned and optimized through training. In a neural network, signals pass through the neurons, weighted summation, and activation functions in the input layer, then propagate through the hidden layers, and finally reach the output layer. This process is called forward propagation, where input data are passed through the network, generating corresponding output results. Backpropagation is a crucial step in training neural networks. It calculates the gradients of the network parameters (weights and biases) with respect to the loss function and updates the parameters in the opposite direction of the gradients. This process utilizes the chain rule to compute the influence of each parameter on the loss function and updates the parameters using a gradient descent algorithm.

In related studies, reference [37] proposed a method to enhance Global Navigation Satellite System (GNSS) positioning by employing deep learning algorithms, utilizing DNNs to learn position corrections based on GNSS measurements. The architecture, using a set-based deep learning approach, accommodates variations in input quantity and order, and a data augmentation strategy is introduced to reduce overfitting. Validation through simulations and real-world data demonstrates improved positioning accuracy compared to baseline methods. Reference [38] proposed the GA-ACO-BPNN model to enhance indoor positioning services through UWB DS-TWR technology. This method addresses the challenge of ranging errors by integrating Genetic Algorithm (GA) and Ant Colony Optimization (ACO) algorithms into a Backpropagation Neural Network (BPNN). Reference [39] proposed a novel indoor positioning system combining Artificial Neural Networks (ANN) with swarm intelligence to overcome the limitations of GPS in indoor environments. Their hybrid algorithm utilizes ANN for indoor location and integrates swarm intelligence for efficient parameter optimization. Experimental results demonstrate significant reductions in search time while maintaining positioning accuracy comparable to exhaustive methods. Reference [40] proposes a DenseNet for calibrating Channel State Information (CSI) to reduce errors in indoor positioning.

Based on the research on relevant positioning error correction methods and the characteristics of neural networks, it can be concluded that neural network-based error correction methods have the following advantages. Neural network-based error correction methods can model and correct more abstract factors that are difficult to mathematically model, without the need to introduce other signal sources. With a fixed scene, coordinates with errors can be mapped to corrected coordinates. Therefore, in this study, in indoor scenarios with only a single 5G signal source and complex electromagnetic environments, this paper proposes the 5G-PECNN deep learning model for correcting errors generated in 5G positioning.

## 3. Preliminaries

The challenge in this study lies in achieving a mapping from positioning coordinates with error information to corrected coordinates without access to crucial information such as angle-of-arrival (AOA) data or propagation time, while being limited to closed-source devices that only provide measured terminal coordinates and true coordinates. To address this challenge, it is necessary to start from the principles of positioning, explore spatial signal characteristics, and understand the relationship between error features and location as well as the relationship with measured coordinates. Subsequently, an error correction model needs to be established to achieve the mapping from positioning coordinates with error information to corrected coordinates.

Firstly, therefore, we analyze the 5G positioning method based on AOA and the error generation model. Next, we introduce the dataset collected from real-world scenarios and then analyze the error characteristics of the dataset based on the error model.

### 3.1. AOA Localization Principles and Error Models

**Figure 1:**
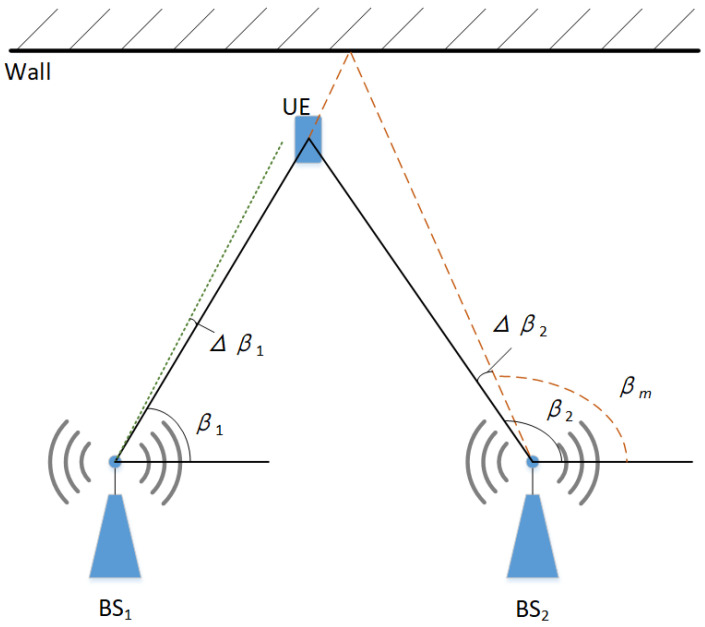
Schematic diagram of AOA localization, where the black line is the normal signal propagation, the green dashed line is the noise interference schematic, and the orange dashed line is the multipath influence schematic.

The AOA positioning technique utilizes the built-in antenna array at the receiving end of the base station, denoted as BSi, to capture the azimuthal angle of the electromagnetic wave transmitted from the user terminal (UE). This information is used to formulate the tangent function equation, as depicted in Figure 1. The coordinates of the base station $BS_{1}$ are $\left(x_{1},y_{1}\right)$, the coordinates of the base station $BS_{2}$ are $\left(x_{2},y_{2}\right)$, and the coordinates of UE are (X,Y). Without considering the noise and the multipath influence, the measured angle of arrival of the base station $BS_{1}$ is $\beta_{1}$, and the angle of arrival of the base station $BS_{2}$ is $\beta_{2}$, which can be obtained by the trigonometric function Equation (1):

(1) $$\tan\beta_{1}=\frac{y_{1}-Y}{x_{1}-X}$$

(2) $$\tan\beta_{2}=\frac{y_{2}-Y}{x_{2}-X}$$

Combining the two equations, the horizontal and vertical coordinates of the user’s UE are obtained as:

(3) $$X\;\;=\;\;\frac{y_{2}-y_{1}+x_{1}\tan\beta_{1}-x_{2}\tan\beta_{2}}{\tan\beta_{1}-\tan\beta_{2}}$$

(4) $$Y\;\;=\;\;\frac{y_{2}\tan\beta_{1}-y_{1}\tan\beta_{2}+\tan\beta_{1}\tan\beta_{2}\left(x_{1}-x_{2}\right)}{\tan\beta_{1}-\tan\beta_{2}}$$

However, in the indoor signal propagation process, it is necessary to consider the influence of noise and multipath propagation on the signal. This leads to errors in the positioning results. Taking the analysis of the relationship between the measured coordinates and the true coordinates as an example of individual noise interference and individual multipath propagation interference, it is assumed that the measured arrival angle $\beta_{1}$ at base station $BS_{1}$ will have an error $\Delta \beta_{1}$ due to noise interference. The multipath signal arrival angle $\beta_{m}$ at base station $BS_{2}$ results in an error $\Delta \beta_{2}$ relative to the true arrival angle $\beta_{2}$. The measured coordinates $X_{measured}$ and $Y_{measured}$ obtained in this way are shown in Equations (5) and (6).

(5) $$X_{measured}\;\;=\;\;\frac{y_{2}-y_{1}+x_{1}\tan\left(\beta_{1}\pm \Delta \beta_{1}\right)-x_{2}\tan\left(\beta_{m}\pm \Delta \beta_{2}\right)}{\tan\left(\beta_{1}\pm \Delta \beta_{1}\right)-\tan\left(\beta_{m}\pm \Delta \beta_{2}\right)}$$

(6) $$Y_{measured}\;\;=\;\;\frac{y_{2}\tan\left(\beta_{1}\pm \Delta \beta_{1}\right)-y_{1}\tan\left(\beta_{2}\pm \Delta \beta_{2}\right)+\tan\left(\beta_{1}\pm \Delta \beta_{1}\right)\tan\left(\beta_{2}\pm \Delta \beta_{2}\right)\left(x_{1}-x_{2}\right)}{\tan\left(\beta_{1}\pm \Delta \beta_{1}\right)-\tan\left(\beta_{2}\pm \Delta \beta_{2}\right)}$$

By comparing Equations (3) and (5), it can be concluded that there is a certain correlation between the true coordinates *X* of the *UE* and $X_{measured}$, and there exists a rather complex mapping relationship. That is, $X_{measured}$ can be expressed as a function of *X*, $\Delta \beta_{1}$, and $\Delta \beta_{2}$, as shown in Equation (7).

(7) $$X_{measured}\;\;=\;\;f\left(X,\Delta \beta_{1},\Delta \beta_{2}\right)$$

In relatively static indoor scenarios, the noise and multipath effects at a particular location are correlated with the position of the terminal, i.e., $\Delta \beta_{1}$ and $\Delta \beta_{2}$ can be expressed as functions associated with *X* and *Y*. Substituting this into Equation (7), we obtain Equation (8).

(8) $$X_{measured}\;\;=\;\;f^{\prime }\left(X,Y\right)$$

Similarly, $Y_{measured}$ can be obtained as shown in Equation (9).

(9) $$Y_{measured}\;\;=\;\;g^{\prime }\left(X,Y\right)$$

Combining Equations (8) and (9), the mapping relationship between ($X_{measured}$, $Y_{measured}$) and (*X*, *Y*) can be obtained as shown in Equation (10).

(10) $$\left(X_{measured},Y_{measured}\right)\;\;=\;\;h\left(X,Y\right)$$

According to Equation (10), the mapping relationship between (*X*, *Y*) and ($X_{measured}$, $Y_{measured}$) can be derived as shown in Equation (11).

(11) $$\left(X,Y\right)\;\;=\;\;p\left(X_{measured},Y_{measured}\right)$$

In theory, the derivation process from Equations (7)–(11) is achievable, and according to Equation (11), it is possible to easily calculate the true coordinates from the measured coordinates. However, in practice, the derivation process from Equations (7)–(11) is extremely complex, and the mapping function of noise with respect to coordinates is nearly impossible to represent accurately with an exact function. In situations where the mapping relationship between the two is known to exist but is challenging to directly express, neural networks excel at solving such problems. By modeling with neural networks, training through regression, and fitting the relationship in Equation (11), an approximation of the mapping relationship shown in Equation (11) can be obtained. This aims to achieve the goal of deducing the true coordinates from the measured coordinates, thus reducing positioning errors.

### 3.2. 5G Indoor Positioning Dataset

**Figure 2:**
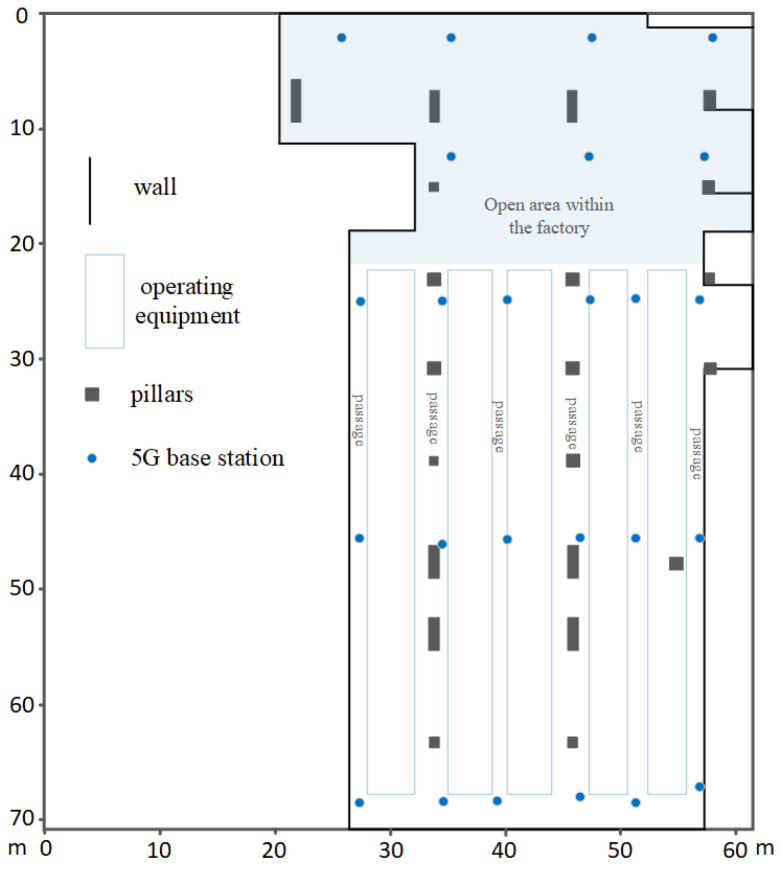
Schematic plan of the plant workspace; the blue point is the 5G base station. All areas are within the factory’s working space. The light-blue area represents the open space inside the factory. The blue rectangle below indicates operational equipment, with an aisle in the middle for pedestrian passage.

The plan view of the working room within the factory area is illustrated in Figure 2, depicting the layout of the 5G base stations. At each location, point within the working area, 5G signal data are collected, and the measured coordinates are determined using the angle-of-arrival (AOA) localization algorithm, as in Equations (5) and (6). Simultaneously, these measured coordinates are compared with the real coordinates, forming the database of AOA-measured and real coordinates. This process results in a dataset comprising 16,300 items.

The study utilizes commercially deployed 5G base stations by Chinese operators, conducting experiments using common smartphones. The 5G base stations are assumed to be approximately 3 m in height. Within the factory premises, open areas are spaced at intervals of 3 to 4.2 m, while aisle areas are spaced at 3 m intervals to avoid equipment and collect data. There are a total of 163 measurement points, evenly distributed throughout the entire factory area. Terminals are placed at the test points, mounted on smartphone stands at a height of approximately 1.5 m above the ground. The sampling frequency is 1 Hz, with 100 samples taken at each sampling point. All sample data include a sampling timestamp, measured terminal coordinates ($X_{measured}$, $Y_{measured}$), and actual terminal coordinates ($X_{real}$, $Y_{real}$).

In direct line-of-sight conditions without any other electromagnetic interference, the positioning error of the terminal obtained through the AOA localization method is less than 0.5 m. However, in real-world scenarios, the positioning error varies depending on the environment at different locations, showing different rates of increase. Please refer to the subsequent discussion for the analysis of positioning errors in each area.

**Figure 3:**
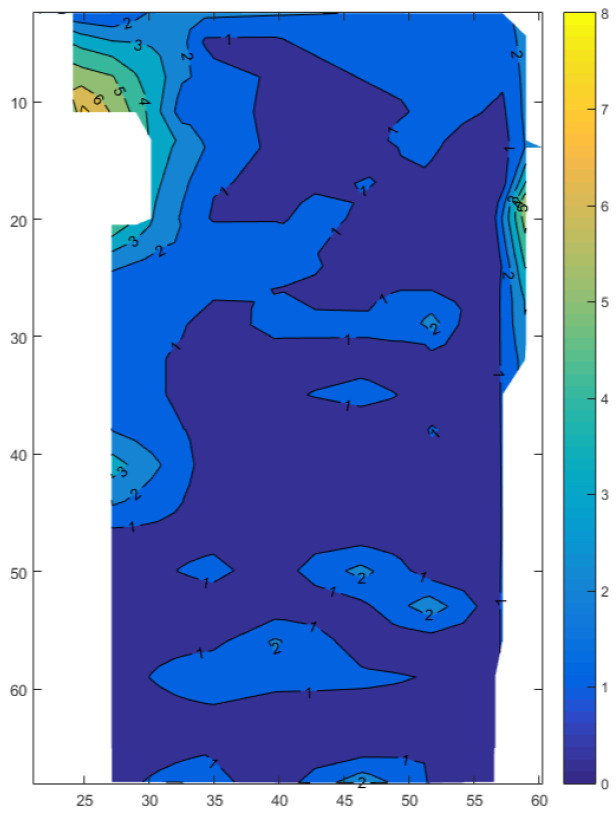
Contour plot of the error of the measurement results at various locations in the indoor work area. The numbers on the contour lines represent positioning errors, and areas with the same color indicate the same range of positioning errors.

We calculate the Euclidean distance as the measurement error according to the real coordinates and the measured coordinates in the dataset and obtain the error contour map of the indoor working area as shown in Figure 3. Figure 3 shows the 2D positioning results, where the horizontal and vertical axes represent the true coordinates inside the factory. The contour lines in the figure indicate the error values of the measured coordinates at corresponding positions.

The horizontal and vertical axes represent the x-coordinate and y-coordinate, respectively, and the contour lines of different colors represent the errors of different values.

From Figure 3, it is evident that positioning measurement errors are more pronounced in the corners of the work area and near the walls. This is primarily attributed to multipath effects and increased noise around the wall corners.

**Figure 4:**
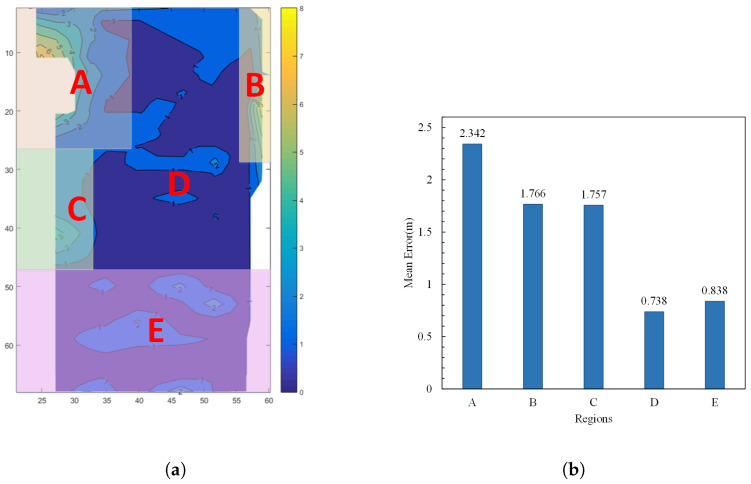
Schematic diagram of partitions and bar chart of average errors. (**a**) Schematic diagram of work area zones. The regions labeled A–E represent five distinct areas delineated in the study. (**b**) Bar chart of average errors in each region.

Based on the features presented in the error contour map, we have divided the entire workspace into five regions: A, B, C, D, and E, as shown in Figure 4a. The average positioning errors for each region are depicted in the bar chart in Figure 4b.

According to Figure 4b, it can be observed that the average positioning errors in regions A, B, and C are significantly higher than in other areas. Particularly, in region A, which is located in the corner of the wall, the signal features are more complex, leading to larger positioning errors under various interferences. In regions B and C, based on the AOA positioning error model, it is known that positioning errors are often caused by factors such as multipath propagation and noise. The proposed model will focus on correcting these error factors.

In the subsequent step, this paper will delve into an analysis of the error sources based on positioning principles. Following this analysis, a novel positioning correction algorithm will be proposed.

## 4. Methods

Regression analysis [41] is a statistical analysis method for determining interdependent quantitative relationships between two or more variables, and the correction from measured to real coordinates is a typical regression problem. Neural networks [39,42,43] have typical nonlinear mapping capabilities to solve regression problems. This section describes the research on the 5G-PECNN algorithm, improvements to the designed model, and details of algorithm optimization methods.

### 4.1. 5G Positioning Error Correction Neural Network

A neural network can be trained and learned from data to establish relationships between input and output variables without the need for an explicit mathematical model. The network has an input layer, an output layer, and several hidden layers. Each layer contains several neurons, and information transfer between neurons is realized through weights and activation functions [44]. The neural network has obvious advantages in solving complex regression prediction problems with discretized data and has good performance in localization error correction [45].

**Figure 5:**
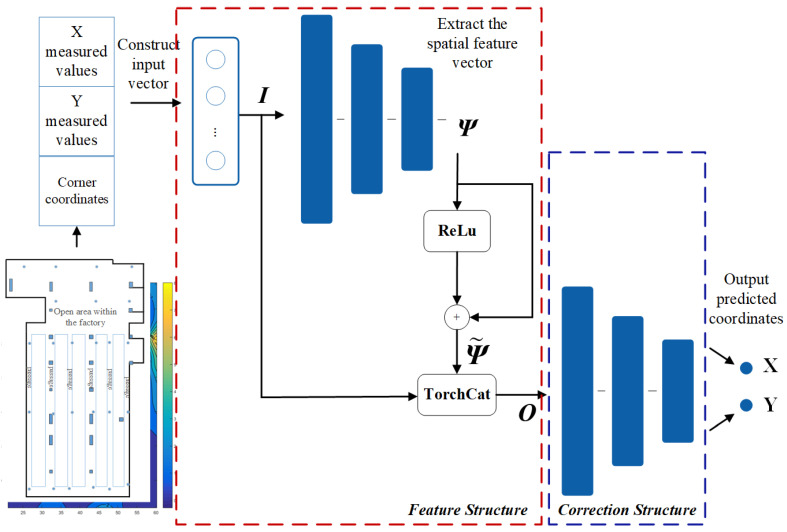
The schematic diagram of the constructed neural network model A. The three connected blue rectangles represent the hidden layers of the network. In the Feature structure, the numbers of neurons in the hidden layers are 16, 8, and 4, respectively, while in the Correction structure, they are 30, 16, and 8, respectively.

Based on the constructed 5G positioning dataset and error characteristics, a neural network model is established as illustrated in Figure 5, referred to as model A. The input layer comprises estimated coordinates and coordinates of corners within the indoor working area. In this input layer, apart from the estimated coordinates, the coordinates of corners are simultaneously fed into the neural network. As indicated in Figure 3, significant noise and multipath effects are observed at the corners and along the walls, resulting in considerable positioning errors in these locations. Therefore, the coordinates of corners are utilized as factors mapping to error-related elements that are inputted into the neural network. This forms a multi-dimensional input vector using both the estimated coordinates and the coordinates of corners.

As shown in Figure 5, the structure within the red dashed box of model A is the spatial signal feature extraction structure. The input tensor is the constructed input tensor I. The three rounded rectangular bars represent hidden layers constructed by neurons, and the neurons in multiple hidden layers are interconnected to form a neural network structure used to extract the spatial feature tensor Ψ. The tensor $\tilde{\Psi }$, obtained through residual propagation, is fused with tensor I to construct tensor O through tensor fusion. Tensor O, serving as the input tensor fused with spatial signal features and error information, is passed into the error correction structure. The error correction model fits the spatial signal features and error information, outputting the corrected coordinates. As shown in Equation (12), where *g*(*x*) represents the correction effect of the correction structure.

(12) x,y=gI||Ψ˜=gI||Ψ+fReLUΨ

In the process of forward propagation, initially, spatial signal characteristics are extracted and learned through a feedforward neural network to construct a spatial signal propagation model. The extracted spatial feature vector undergoes residual operations and is fused with the input estimated coordinates, creating a tensor fusion. This fused tensor is then fed into the error correction network to analyze and learn from the fused spatial signal features and the contained error information, subsequently correcting the estimated coordinates. The output layer represents the corrected user terminal coordinates. During the training phase, the output layer comprises the actual coordinates of the user terminal, aiming to establish a correction model from the estimated coordinates to the real coordinates.

**Figure 6:**
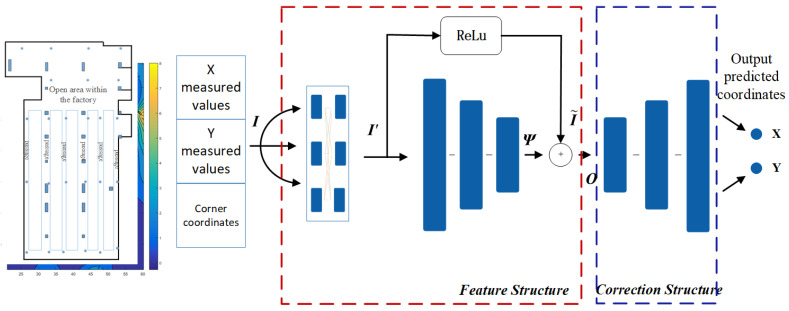
The schematic diagram of the constructed neural network model B. The three connected blue rectangles represent the hidden layers of the network. In the Feature structure, the numbers of neurons in the hidden layers are 16, 8, and 4, respectively, while in the Correction structure, they are 6, 8, and 16, respectively.

Model A employs two regular pyramid structures, while model B incorporates two symmetric pyramid structures. A comparison of their effectiveness is conducted to evaluate their respective performance. Based on the improvement of model A, the optimized model B is obtained, as illustrated in Figure 6. Through repeated studies, it was found that by extracting partition features from the input tensor I to obtain tensor $I^{\prime }$, performing residual propagation in one direction to obtain tensor $\tilde{I}$, and passing it into a hidden layer to extract spatial feature tensor Ψ in another direction, the tensor Ψ is fused with tensor $\tilde{I}$ to construct tensor O. Tensor O, as the input vector fused with spatial signal features and error information, is passed into the error correction structure. The error correction model fits spatial signal features and error information, outputting the corrected error coordinates. In the correction structure, inverting the number of neurons in the hidden layer can better expand the fused spatial signal features and error information, forming correction factors to correct coordinates containing errors, resulting in a better correction effect. As shown in Equation (13), where *g*(*x*) represents the correction effect of the correction structure.

(13) x,y=gI˜||Ψ=gfReLUI′||Ψ

The ReLU function is selected as the activation function during training (Equation (14)) to reduce the model computation and avoid overfitting and gradient disappearance

(14) fReLUx=max0,x

The MSE (mean squared error) function was chosen as the loss function of the model (Equation (15)).

(15) $$MSE\;\;=\;\;\frac{1}{n}\sum_{n=1}^{n}{\left(y_{i}-{\hat{y}}_{i}\right)}^{2}$$

In Equation (15), *n* represents the number of samples in the testing set. $y_{i}\left(i=1,2,\cdots ,n\right)$ denotes the actual values for the ‘*i*-th’ sample, and ${\hat{y}}_{i}\left(i=1,2,\cdots ,n\right)$ represents the predicted values for the ‘*i*-th’ sample. The mean squared error (*MSE*) is a measure where a smaller value indicates that the model’s prediction errors are also smaller.

After repeated experimental validation to adjust the model structure, the 5G localization error correction network is obtained.

### 4.2. Adaptive Global Gradient Descent Algorithm

**Figure 7:**
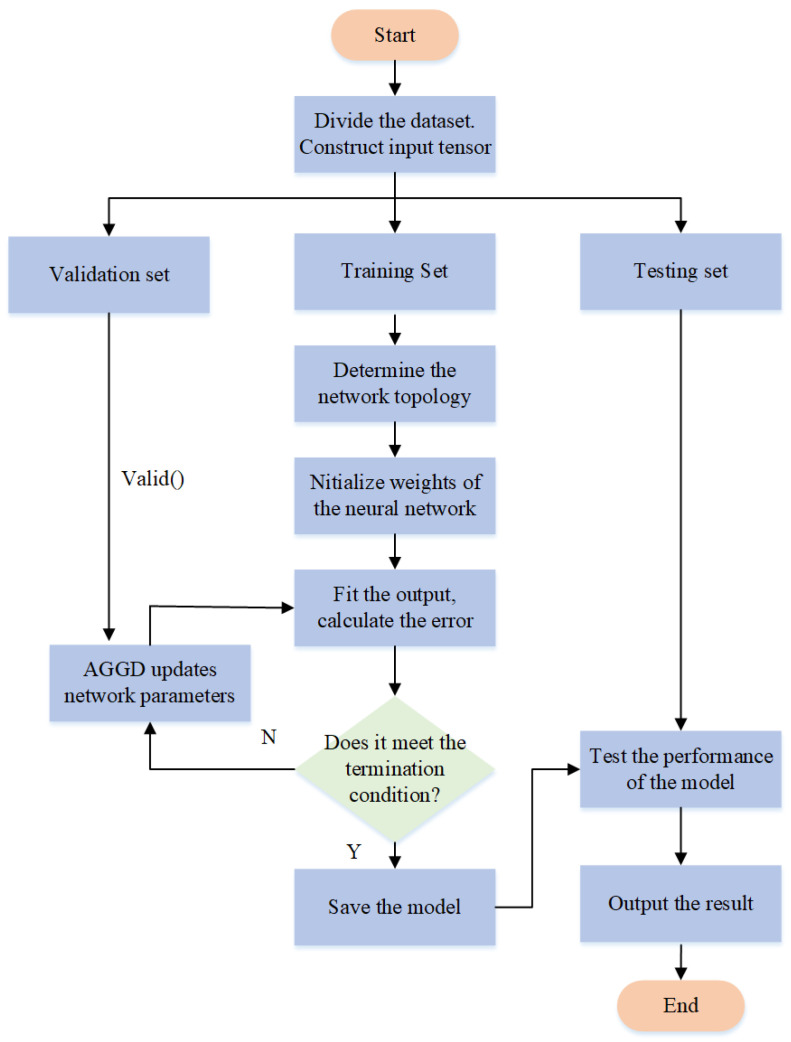
Algorithm Flowchart of the 5G Positioning Error Correction Neural Network based on an Adaptive Global Gradient Descent Algorithm.

In general, the solution for neural network models involves descending along the negative gradient direction [36,46]. Due to the random initialization of initial weights and thresholds, neural network models are prone to becoming stuck in local minimums and failing to achieve global optimal solutions. This often leads to poorer predictive performance, larger errors, and, in our experimental environment, an inclination towards local minimums resulting in outputting local mean coordinates. To prevent the model from being trapped in local optimal solutions, this paper introduces a Positioning Error Correction Neural Network for 5G (5G-PECNN) based on an Adaptive Global Gradient Descent (AGGD) algorithm. The algorithm’s workflow is depicted in Figure 7.

Firstly, the dataset is randomly shuffled, and 20% is randomly selected as the test set, 20% as the validation set, and 60% as the training set. The training set is used for model training. During the training process, the model’s network topology is determined, the neural network weights are initialized, and then the iterative training process begins. In each epoch, the pre-built model predicts output results, compares them with the ground truth to calculate the output loss. The training continues until the termination conditions are met. The AGGD algorithm is employed to update network parameters, with the validation set used to verify the model’s performance and prevent overfitting. Once the termination conditions are met, the trained model is saved, and the test set is used to evaluate the model’s performance, producing the test results.

This paper proposes using a network parameter updating method based on AGGD, replacing the direct loss backpropagation and parameter updating method in neural networks, effectively avoiding the model from falling into local mean-value minimum solutions. The network parameter updating method based on AGGD is depicted in Algorithm 1.
**Algorithm 1:** pseudo code of AGGD**1**
Tensor $W_{0}$, Tensor $L_{0}$**2****Parameters:** $W_{0}$: Weight Parameters of Neural Network Nodes in the Previous Training Epoch; $L_{0}$: Loss Values of the Neural Network for the Previous n Training Epochs.**3****Operation:** The algorithm is based on the Pytorch framework, please refer to Pytorch [47] for related torch operations.**4****Output: W**: Weight Parameters of Neural Network Nodes in this Training Epoch.**5****def** AGGD(self, $W_{0}$,$L_{0}$):**6**  # Initialization**7**  **W** = $W_{0}$**8**  *optimizer = torch.optim.Adam(model.parameters(), lr = 0.0004)***9**  ***yPred*** = *model(x)*  #Predict output using previous generation weights**10**  *l = torch.nn.MSE(yPred, yReal)***11**  $yPredVariance\;\;=\;\;\frac{1}{mn}\sum_{i=1}^{n}{\sum_{j=1}^{m}\left(y_{i}Pred-Mean\left(\mathrm{yPred}\right)\right)}^{2},\left(yPred_{i}\subset \mathrm{yPred}\right)$**12**  ***L*** = $L_{0}$.*append(l)***13**  $Lvariance\;\;=\;\;\frac{1}{n}\sum_{i}^{n}{\left(l_{i}-Mean\left(L\right)\right)}^{2},\left(l_{i}\in L\right)$**14**  # Update network weights**15**  flag=count≥150&l>0.006&Lvariance<0.1&yPredVariance<0.2**16**  *if flag*:**17**    $W\;\;=\;\;torch.random.initWeight\left(W_{0}\right)\;\;while\;\;W\;\;=W_{0}$**18**    *count = 0***19**    *L = []***20**  *else*:**21**    *optimizer.zero_grad()***22**    *loss.backward()***23**    *count += 1***24**    *valid()*

In general scenarios, optimization gradient descent algorithms like AdaGrad and Adam can reduce the probability of falling into local optima to some extent. However, in different scenarios, it is still possible to encounter local optima, necessitating the consideration of specific situations in the studied scenario to help escape local optima and find global optima. In the context of this paper, during the training process, there is a certain probability of falling into the mean solution of certain coordinates at some locations, causing the loss value to become trapped in a local optimum. Therefore, it is necessary to first determine whether a local optimum has been reached and then escape it.

In this scenario, it is determined that when the training iteration is greater than or equal to a certain threshold, the loss value is greater than a certain threshold, and the variance of the loss value is less than a certain threshold, it represents a stable situation where the loss value reaches a local minimum, indicating convergence to a local minimum. At this point, if the variance of the inferred coordinates (yPred) is less than a certain threshold, it indicates that the algorithm has converged to a local minimum. Here, *l* represents the loss value for each epoch, yPred represents the inferred coordinates, yPredVariance represents the variance of the inferred coordinates, Lvariance represents the variance of the loss value, count represents the iteration count, and flag indicates whether a local minimum has been reached.

When it is determined that a local minimum has been reached, the model escapes from the local optimum by randomly shuffling the network weights, leading to an increase in the loss value. If the condition for reaching a local minimum is not met, the Adam gradient descent algorithm is employed.

Therefore, existing gradient descent algorithms focus more on optimizing the descent process during gradient descent to achieve rapid descent. The method proposed in this paper is based on the special characteristics of the scenario, by adding criteria to determine whether a local optimum has been reached. When the existing algorithm fails to escape from the local optimum, it shuffles the model weights and then searches for the global optimum using the existing gradient descent algorithm. The improvement to existing gradient descent algorithms lies primarily in adding criteria for determining whether a local optimum has been reached, which are suitable for specific scenarios, and escaping from local optima by shuffling model weights.

## 5. Results and Discussion

Based on the dataset prepared in Section 3, the performance of the designed 5G-PECNN algorithm will be tested.

### 5.1. Experimental Environment and Dataset Division

The dataset containing 16,300 data points of measured and real coordinates is randomly disrupted, 20% of it is selected as a test set to test the application effect of the algorithm, and the rest is used as a training set to train the 5G-PECNN model. A total of 25% of the training set is used as a validation set to validate the iterative effect of the model during the training process and is not involved in the gradient descent. The hardware environment for training and testing includes an Intel i7-11800H CPU, 32 GB of memory, and an RTX3060 Laptop GPU, all running on the Windows 10 operating system. The software environment comprises Python version 3.8.17, PyTorch version 1.8.2, and CUDA version 11.1.

### 5.2. Test Results and Analysis

The Euclidean distance from the coordinates of the predicted point to the coordinates of the real point is chosen as the error evaluation standard when the test results are evaluated, i.e., the smaller the Euclidean distance represents the closer the predicted results are to the real coordinates, and the smaller the error is. The Euclidean distance is shown in Equation (16).

(16) $$d=\sqrt{\sum_{i=1}^{2}{\left(x_{i}-y_{i}\right)}^{2}}$$

where $x_{i}$ denotes the predicted coordinates and $y_{i}$ denotes the true coordinates.

**Figure 8:**
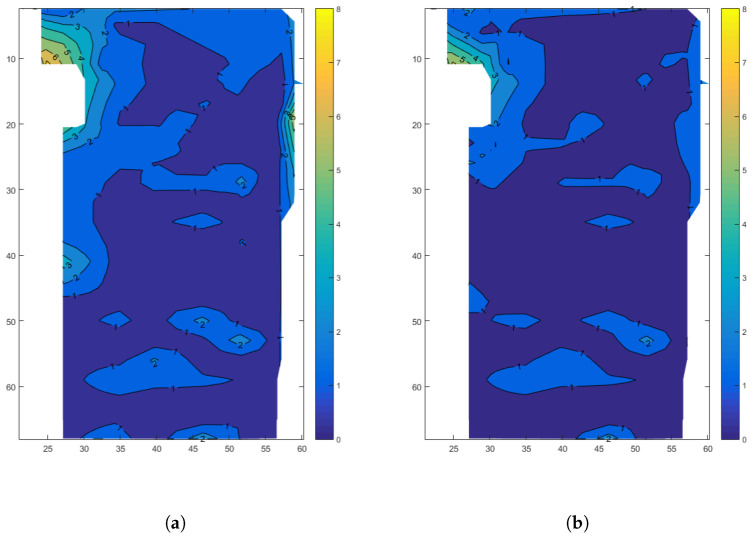
Overall test effect diagram. (**a**) Original error distribution. (**b**) Corrected error distribution.

Its overall position mapping error distribution is shown as Figure 8, with the original data on the left and the error-corrected data of the 5G-PECNN model B on the right. The mean values of the errors before and after the error correction are 1.2 m and 0.934 m, respectively.

Comparing the original error distribution and the corrected error distribution in Figure 8, it can be observed that significant positioning errors occur around the corners and near the walls within the working area. These errors primarily result from multipath effects and increased noise around the corners. In these specific areas, the 5G-PECNN (5G positioning error correction neural network) model B shows a significant error correction effect.

**Figure 9:**
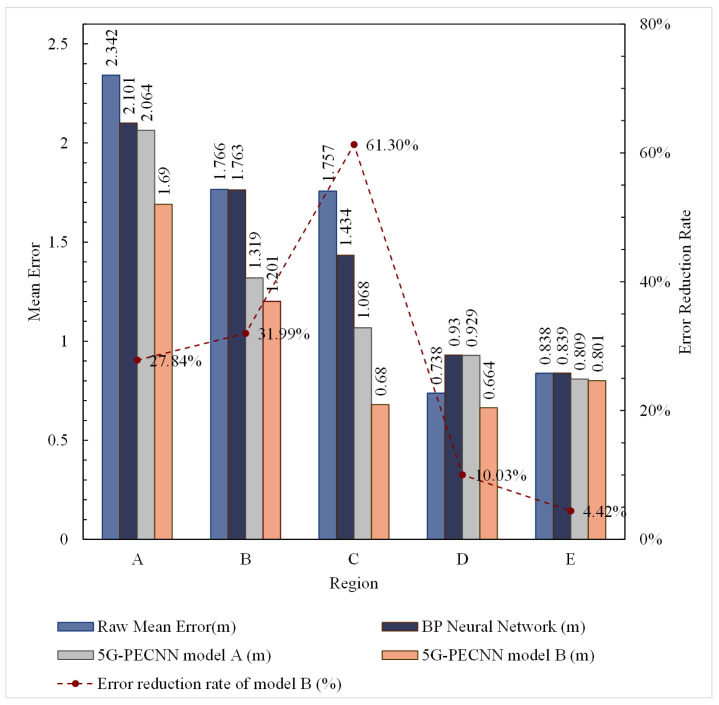
Performance comparison chart for different models in different regions.

To further analyze the correction performance of 5G-PECNN, we evaluated the correction effect in each area according to the workspace division shown in Figure 4a. The results are presented in Table 1 and Figure 9.

Based on Table 1 and Figure 9, the traditional backpropagation neural network (BPNN) only exhibits some correction effects in areas A and C, but its impact is not significant in other regions. It is evident that the 5G-PECNN model A exhibits a more notable positioning correction effects in Regions B and C. It shows a certain level of correction in Region A, but the correction effect in Region E is not distinct. Moreover, the error correction in Region D has a negative impact. Analysis of Regions D and E indicates that these areas are vast open spaces with minimal signal noise and fewer multipath interferences. Due to fewer factors contributing to positioning errors that can be corrected by model A, its efficacy in these regions is suboptimal, leading to ineffective or even increased positioning errors. After excluding the data from Region D, the average error decreased by 37.4% after the comprehensive positioning error correction in other regions. Consequently, model A demonstrates effective correction results in areas with noise and multipath interferences like corners and walls.

In response to the performance of model A, model B has made improvements to the feature structure of model A. Firstly, the input vector was optimized for extracting regional feature tensors. The extracted tensor was then fused with the spatial signal feature tensor output by the feature structure. This allows for better fitting of spatial signal features. In the correction structure, inverting the number of neurons in the hidden layer can better expand the fused spatial signal features and error information, forming correction factors to correct coordinates containing errors, resulting in a better correction effect.

As shown in Table 1 and Figure 9, model B achieved significant error correction effects in areas A, B, and C. The error reduction rates are 27.84%, 31.99%, and 61.30%, respectively. For regions D and E, which originally had smaller average errors, model B also achieved some correction effects without expanding the errors. Constrained by the inherent performance of 5G signals in achieving positioning accuracy, the positioning accuracy corrected by the 5G-PECNN constructed by model B is already sufficiently excellent.

In conclusion, the 5G-PECNN constructed by model B has achieved the best error correction results.

## 6. Conclusions

In this paper, the advantages and trends of using 5G signals for positioning in indoor environments with signal obstruction, such as GPS, are first introduced. The paper analyzes the problems in 5G positioning and presents the practical issues addressed in this study. Subsequently, the research explores the principles of 5G positioning, 5G NR positioning, related studies on wireless signal positioning error correction, and relevant methods for positioning error correction. Based on the analysis above, the paper starts from the principles of 5G angle-of-arrival (AOA) positioning and analyzes the impact of noise and multipath interference in the spatial propagation of 5G signals on positioning results. Addressing the resulting positioning errors, the paper introduces a 5G Positioning Error Correction Neural Network (5G-PECNN) based on an Adaptive Global Gradient Descent Algorithm. Validation of the 5G-PECNN’s correction effectiveness was conducted using a dataset constructed from data collected in the working area. Experimental results demonstrate that the 5G-PECNN exhibits effective correction in positioning results, particularly in areas with noise and multipath interferences such as corners and walls. In areas with significant multipath interference and high signal noise, the positioning errors have been reduced by 27.84% in corners and by 31.99% and 61.30% along the walls. The corrected positioning results of 5G-PECNN approach the accuracy of 5G positioning in an interference-free environment.

However, this method has certain limitations. Since this method focuses on correcting the coordinates output by closed-source devices that cannot obtain signal propagation time, angles, and other information, the correction effect is subject to certain restrictions. In future research, open-source devices will be used in combination with signal geometric information, signal strength information, etc., to correct positioning coordinates, aiming to achieve higher accuracy.

## Figures and Tables

**Table 1:** 5G-PECNN partition error correction effect.

	A	B	C	D	E
Raw mean error (m)	2.342	1.766	1.757	0.738	0.838
BP Neural Network (m)	2.101	1.763	1.434	0.930	0.839
5G-PECNN model A (m)	2.064	1.319	1.068	0.929	0.809
**5G-PECNN model B (m)**	**1.690**	**1.201**	**0.680**	**0.664**	**0.801**

## Data Availability

The data used to support the findings of this study are included in this article.

## Abbreviations

The following abbreviations are used in this manuscript:

5G	5th-Generation Mobile Communication Technology
GPS	Global Positioning System
GNSS	Global Navigation Satellite System
5G NR	5th-generation New Radio
TOA	Time of Arrival
TDOA	Time Difference of Arrival
AOA	Angle of Arrival
RSSI	Received Signal Strength Indication
CSI	Channel State Information
RSRP	Reference Signal Received Power
RSRQ	Reference Signal Quality
5G-PECNN	Positioning Error Correction Neural Network for 5G
AGGD	Adaptive Global Gradient Descent
BP	Backpropagation Neural Network
ReLU	Rectified Linear Unit
MSE	Mean squared error
